# Durability Investigation on CFRP Strengthened Cementitious Materials in Cold Region

**DOI:** 10.3390/polym14112190

**Published:** 2022-05-28

**Authors:** Wei Li, Wenchao Liu, Wenyuan Xu, Yongcheng Ji

**Affiliations:** School of Civil Engineering, Northeast Forestry University, Harbin 150040, China; alice12383@126.com (W.L.); l3268929945@126.com (W.L.)

**Keywords:** CFRP (Carbon Fiber Reinforced Polymer), cementitious materials, salt-freeze coupling environment, finite element analysis, durability

## Abstract

Epoxy resin, CFRP (Carbon Fiber Reinforced Polymer) sheet, and concrete flexural specimens are selected to study the durability of carbon fiber strengthened cementitious materials in a cold region. Two exposure environments, chloride immersion and salt-freeze coupling, are set up, and the mechanical deterioration is discussed utilizing a microscopic observation mechanical test and finite element analysis. The damage to the epoxy resin, CFRP sheet, and concrete exerts a more severe performance degradation in the salt-freeze coupling environment when compared with the chlorine salt immersion environment. The freeze–thaw action destroys the bonding surface of CFRP and concrete based on the microscope observation. The flexural strength of the specimens strengthened with CFRP is 3.6 times higher than that of the specimens without CFRP, while the degradation rate is only 50%. These observations show that the strengthened CFRP effectively improves the cementitious material’s flexural performance in the cold region. The finite element model of epoxy and CFRP subjected to chloride immersion and salt-freeze coupling environment is established. The degradation formula of bond performance between CFRP and concrete is proposed. In addition, the flexural mechanical numerical model is established with and without CFRP strengthened concrete, respectively. Research results provide a technical reference for applying CFRP reinforced cementitious materials in a cold region.

## 1. Introduction

Concrete buildings are simultaneously subjected to the coupling effect of chloride erosion and freeze–thaw cycle (abbreviated to salt-freeze coupled environment) in the marine environment of the cold region. In the salt-freeze coupling environment, concrete buildings deteriorate seriously, making it challenging to ensure their performance and safety. The actual service life of buildings is far from reaching the designed service life [1,2]. The chemical properties of CFRP in a chloride salt environment are relatively stable, and CFRP bonded with concrete can partially prevent the chemical reaction between concrete and chloride salt. Thus, the durability and bearing capacity of a concrete structure is also improved and achieved to strengthen and protect the original damaged concrete structure [3,4]. Toutanjiet et al. [5] studied the effect of carbon fiber on the mechanical properties of cement paste composites. Adding polyacrylonitrile-based carbon fiber to the cement paste matrix can significantly improve the tensile and bending properties of the composites.

The durability of CFRP reinforced concrete structures is influenced by the following factors: the durability of concrete materials, the durability of epoxy resin, the durability of CFRP, and the durability of the interface between CFRP and concrete. However, most researchers only discussed the durability of the above objects in a single chloride salt immersion environment or a single freezing-thawing environment. Therefore, limited research focus on a comprehensive study of the coupling effect of influencing factors [6].

The carbon fiber sheet is presoaked with epoxy resin adhesive on both sides and bonded along the tensile direction for the concrete structure strengthened with CFRP. The CFRP and concrete are stressed together, giving full play to the compressive performance of the concrete and the high tensile performance of the CFRP. This improves the bearing capacity of the concrete structure and achieves the purpose of strengthening the original concrete structure. The factors influencing stress transfer between carbon fiber sheets and concrete include the interface bonding performance between CFRP and concrete and the mechanical properties of the epoxy resin itself. Therefore, it is necessary to study their durability in a chloride environment. Lu et al. [7] studied the durability of epoxy resin adhesive in the dry–wet cycle environment of chloride solution. Results show that chloride salt is deposited on the surface of the epoxy resin adhesive sheet. After 360 dry–wet cycles, the tensile strength, tensile elastic modulus, and ultimate tensile strain of epoxy resin adhesive sheet decreased by 27.8%, 3.2%, and 64.8%, respectively. Sousa et al. [8] investigated the durability of epoxy resin adhesive in a chloride solution. The water absorption and dynamic mechanical analysis (DMA) were performed. The results show that the diffusion of erosion medium in the sheet does not follow Fick’s second law, and the bending and shear properties show plastic characteristics. Rudawska [9] discussed the durability of epoxy resin adhesive in different concentrations of chloride solution, and a digital microscope and the axial compression test were carried out. The compressive strength of the sheet decreases with the increase of chloride concentration under the same soaking cycle. Ascione et al. [10] tested the durability of two commonly used epoxy resin adhesives (SikaDur30 and Araldite) in a seawater immersion environment. The thermal analysis and fracture energy of epoxy resins was evaluated using the flexibility beam method. The results showed that the mechanical properties of epoxy resin degraded more seriously in saline solution than in tap water solution.

CFRP itself has a tremendous advantage of tension, which can improve the bearing capacity of the concrete structure. Therefore, it is necessary to study the durability of CFRP chloride salt and salt-freeze coupled environment, respectively. Hong et al. [11] studied the durability of CFRP sheets under the coupling action of seawater immersion and continuous bending load. Test results show that chloride salt is deposited on the epoxy resin adhesive surface, located in the out-layer of CFRP. Furthermore, the seawater immersion environment has more severe degradation of the tensile properties of CFRP sheets when compared with the distilled water immersion environment. Xie et al. [12] evaluated the durability of CFRP in the dry–wet cycle environment of chloride solution when subjected to 0, 60, 120, 240, and 360 dry–wet cycles, respectively. The results show that the mechanical properties of the CFRP sheet decrease slightly due to the hydrolysis of the epoxy resin matrix, resulting in the damage of the interface between carbon fiber and epoxy resin matrix. Cruz et al. [13] used Fourier transforms infrared spectroscopy (FTIR) to study the chemical characterization of the CFRP sheet in a seawater immersion environment, followed by dynamic mechanical analysis (DMA). Results show that the mechanical properties of CFRP sheets do not change significantly after one year of immersion.

The durability of CFRP reinforced cementitious materials also needs to be further investigated. Nasser et al. [14] studied the durability of reinforced concrete beams strengthened with CFRP in chloride solution. Four-point bending was performed to evaluate the load-displacement relationship, strain, failure mode, ductility, and stiffness. The results show that CFRP improves the ultimate bearing capacity of the beam. A reduction coefficient of flexural strength of CFRP reinforced concrete in a chloride environment is proposed. Choi et al. [15] studied the durability of concrete beams strengthened with CFRP in chloride solution. It is found that the flexural strength of beam specimens decreases with environmental exposure, and the effect of the chloride environment on the bonding performance between concrete and CFRP is evaluated. The durability of reinforced concrete beams strengthened with prestressed CFRP in the dry–wet cycle environment coupled with chloride solution was investigated [16]. It is found that the dry–wet cycle cause loss of prestressing of CFRP, and the prestress is decreased by about 15% after 60 days of exposure. Zhang et al. [17] discussed the effect of freeze–thaw damage on chloride ion penetration into concrete and reinforcement corrosion. The chloride ion diffusion coefficient increases linearly with freezing and thawing times, and the propagation speed of steel corrosion is significantly accelerated. Yu et al. [18] studied the axial performance of concrete strengthened with CFRP under different environments. The CFRP has good corrosion resistance and effectively improves the strength and ductility of specimens. Yang et al. [19] developed the mechanical model of the concrete bridge deck strengthened with CFRP. The numerical simulation results agree with the test results, and the bridge deck makes full use of the high tensile and corrosion resistance of CFRP. Castaldo et al. [20] studied the deterioration of chloride on reinforced concrete structures. The degradation effects of chloride on reinforcement, concrete, and the bonding surface between reinforcement and concrete were discussed. In addition, the model of chloride ion penetration into concrete is established based on Fick’s second law, and the service life is evaluated. Castaldo et al. [21] proposed an additional safety factor based on failure mode, and the overall design resistance related to experimental uncertainty was evaluated. Haukaas et al. [22] proposed a new method to deal with model uncertainty in finite element analysis. An extended model formula is developed based on the comprehensive information database to evaluate laboratory testing, error estimation, and accurate numerical solutions. Gino et al. [23] discussed the influence of experimental uncertainty on the statistical estimation of uncertain random variables of the resistance model. The uncertain resistance model is characterized by comparing experimental results and numerical results, and the cognitive uncertainty is quantified.

The durability of epoxy resin, CFRP sheet, and cementitious materials specimens is investigated in this study. Two corrosive environments are set up: the chlorine salt immersion and the salt-freeze coupling environment. The damage degree of the exposed environment to the above specimens is investigated by microscope observation. The tensile properties of epoxy resin and CFRP sheet (including the ultimate tensile strain of tensile strength and stress–strain curve) are measured by tensile test, and the degradation of the tensile properties of the sheet in the exposed environment is discussed. The flexural properties (including flexural strength, deflection, and load-deflection curve) of specimens subjected to bending are measured by a four-point flexural tension test. In addition, the degradation law of flexural properties of specimens subjected to bending is discussed. A finite element model is proposed to analyze the internal force distribution characteristics, failure modes, and mechanical properties.

## 2. Materials and Methods

### 2.1. Epoxy Resin Adhesive and Carbon Fiber Cloth

**Figure 1:**
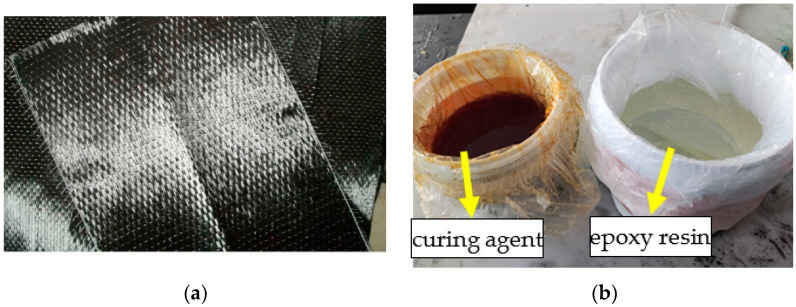
Carbon fiber sheet and epoxy resin adhesive and: (**a**) carbon fiber sheet and (**b**) epoxy resin adhesive.

A unidirectional carbon fiber sheet with a thickness of 0.167 mm is used in the test, as shown in Figure 1a. Carbon fiber can be prepared by blending polyacrylonitrile (Pan) and lignin and then by a wet-spinning process, and it is often used in concrete engineering reinforcement. The CFRP bonding adhesive is divided into A and B components, including a curing agent and epoxy resin (Figure 1b). After 7 days, the curing degree of epoxy resin is 95%. The material properties are shown in Table 1. The manufacturer of epoxy resin and carbon fiber cloth is Shanghai Jingdong Construction Technology Co., Ltd. (Shanghai, China).

**Figure 2:**
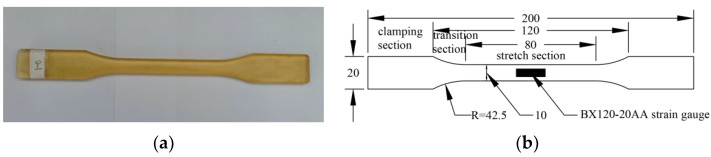
Epoxy resin adhesive sheet: (**a**) sheet and (**b**) size and position of strain gauge.

**Figure 3:**
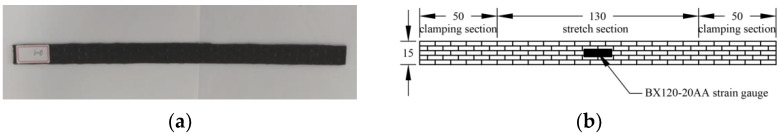
CFRP sheet: (**a**) sheet and (**b**) size and position of strain gauge.

The mix ratio of epoxy resin and curing agent is 2:1, and curing for 7 days at room temperature of 25 °C. The epoxy specimen size and strain gauge layout point are shown in Figure 2. Similarly, the carbon fiber is cut into 250 mm in length and 200 mm in width, then brushed with epoxy resin adhesive on both sides to constitute a CFRP sheet, as shown in Figure 3.

### 2.2. Concrete

**Figure 4:**
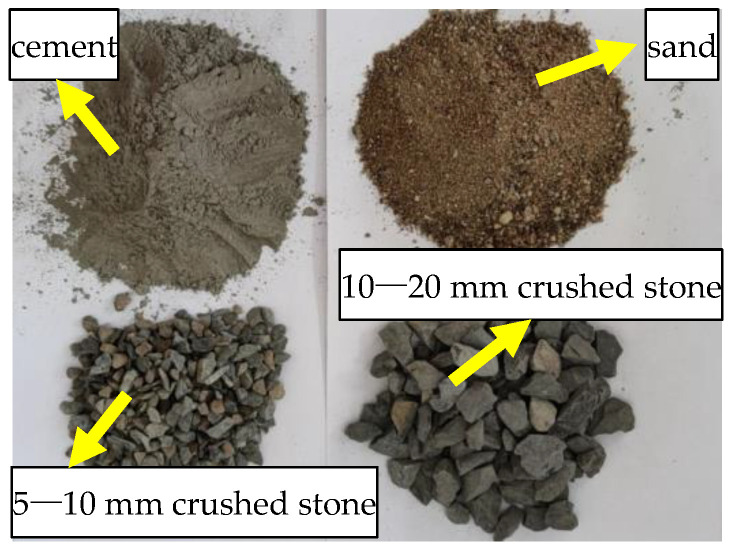
Concrete composition materials.

Concrete design strength is 30 MPa in this test, and its composition materials include water, cement, sand, and gravel, as shown in Figure 4. Table 2 shows the concrete mix design, and the ordinary Portland cement is selected for cement. Fine aggregate is medium sand with a modulus coefficient of 2.4. Coarse aggregate is gravel, of which a particle size of 5~10 mm accounted for 30% and 10~20 mm accounted for 70%.

**Figure 5:**
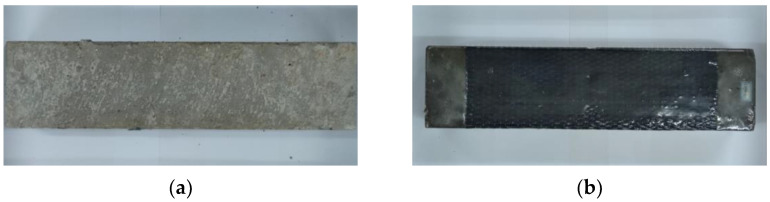
Concrete flexural specimen: (**a**) unreinforced specimen and (**b**) reinforced specimen.

**Figure 6:**
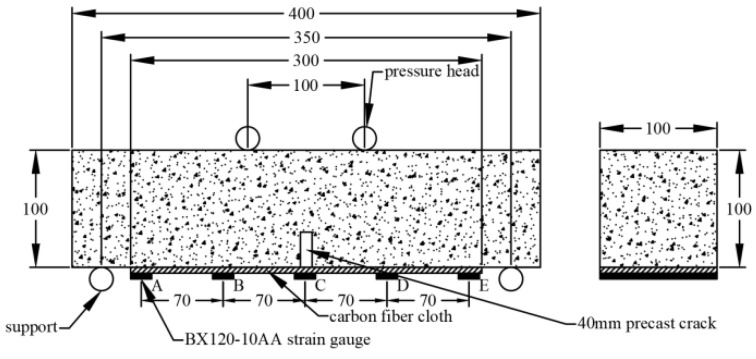
Flexural testing set up.

The unreinforced and CFRP reinforced concrete prism specimens are selected, and the dimension of a specimen is 100 mm width by 100 mm height by 400 mm length. A notch of 30 mm height is prefabricated on the midspan’s tension side to guarantee bending failure, and a rectangle shape of 100 mm × 300 mm CFRP strip bonded on the tensile side of CFRP reinforced concrete specimen, as shown in Figure 5a,b. The flexural performance and durability of CFRP reinforced concrete are discussed. Figure 6 shows the four-point loading position, and a strain gauge is bonded on the surface of CFRP to measure the strain variation during loading development.

### 2.3. Exposure Environment

**Figure 7:**
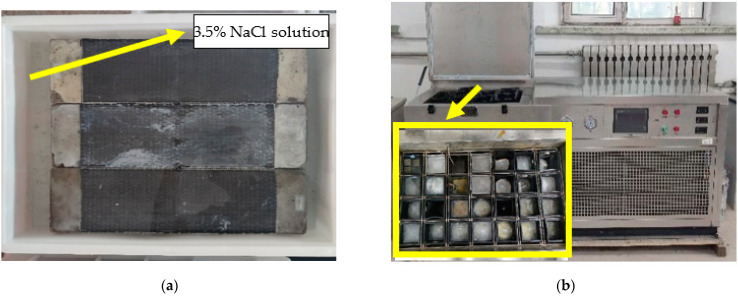
Exposure environment: (**a**) chlorine salt immersion environment and (**b**) salt-freeze coupled environment.

**Figure 8:**
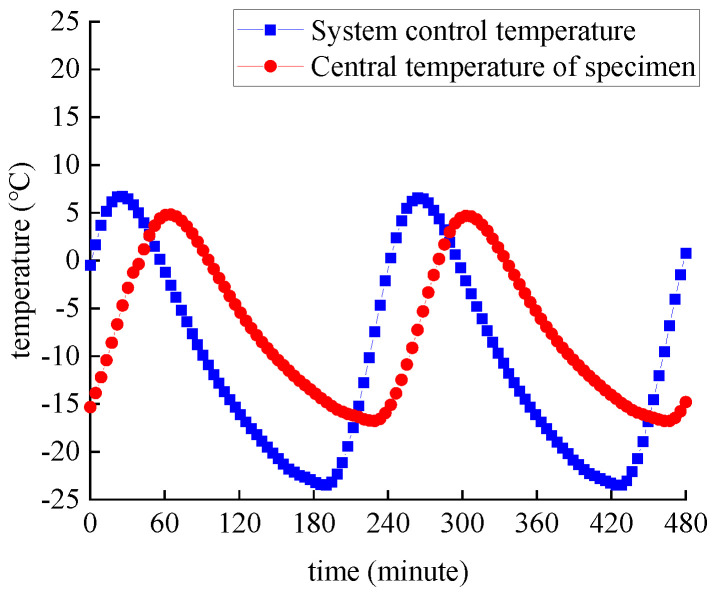
Temperature curve.

There are two kinds of exposure environments: chlorine immersion and salt-freeze coupling effect, respectively. Figure 7a shows the specimen immersed in NaCl solution with a mass concentration of 3.5% at room temperature of 25 °C; that is, in the chlorine-salt immersion environment. As shown in Figure 7b, the freeze–thaw cycle test is carried out using the quick-freezing method, with a freeze–thaw cycle period of 4 h. The freezing–thawing medium is NaCl solution with a mass concentration of 3.5%, which is the salt-freeze coupling environment. The central temperature of specimens and the inner freezing and thawing machine are monitored, and the temperature curve variation is shown in Figure 8.

The epoxy resin, CFRP sheet, and concrete specimens are grouped and numbered when subjected to various environments and duration. The definition of numbering information is shown in Table 3 and Table 4. The serial number of Carbon Fiber Reinforced Polymer is abbreviated to CFRP, and the epoxy resin is abbreviated to EP. Similarly, the reinforced concrete specimen is abbreviated to RF, and the unreinforced concrete specimen is abbreviated to URF. For environmental effect, no deterioration is abbreviated to ND, chlorine salt immersion environment is abbreviated to SI, and salt-freezing coupled environment is abbreviated to SF.

### 2.4. Microscopic Observation

**Figure 9:**
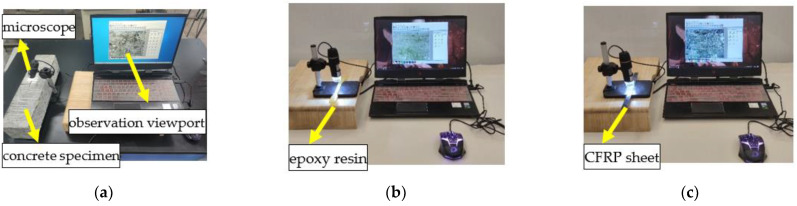
Microscopic observation: (**a**) concrete flexural specimen; (**b**) epoxy resin sheet; and (**c**) CFRP sheet.

A digital microscope is used to observe the damage degree of epoxy resin, CFRP sheet, and concrete specimens, respectively. The typical epoxy resin of EPND, EPSI-400, and EPSF-100 is selected to observe its surface deterioration. Similarly, CFRPND, CFRPSI-400, and CFRPSF-100 are selected for CFRP; URFND, URFSI-400, and URFSF-100 are selected for the unreinforced specimen; and RFND, RFSI-400, and RFSF-10 are selected for the reinforced specimen, as shown in Figure 9.

### 2.5. Mechanical Test

**Figure 10:**
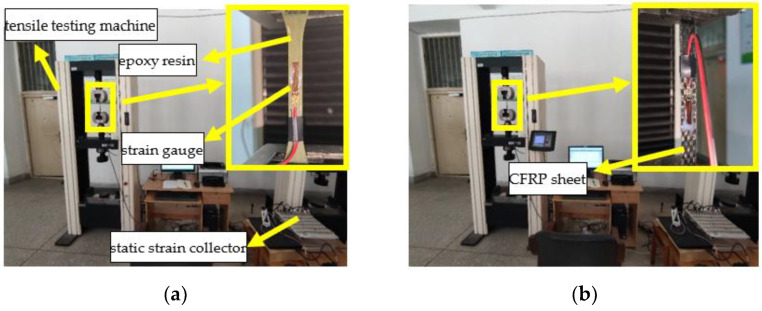
Tensile mechanical test: (**a**) epoxy resin and (**b**) CFRP sheet.

In order to investigate the mechanical performance deterioration in a corrosive environment, tensile tests are carried out on epoxy resin and CFRP sheets. The tensile test process is composed of mechanical control and data acquisition system, and the loading rate is 2 mm/min, as shown in Figure 10.

**Figure 11:**
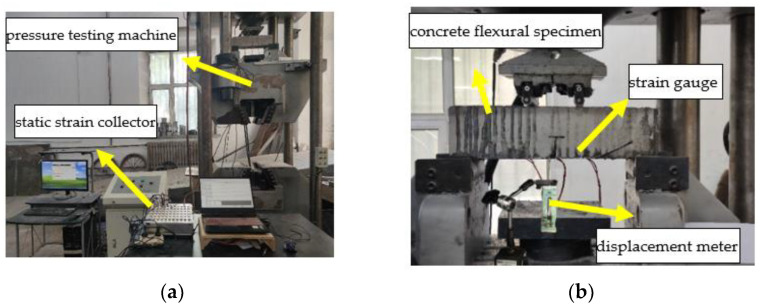
Bending mechanical test: (**a**) load general setting and (**b**) detail data collection setting.

Figure 11 shows the four-point flexural tension test for concrete specimens concerning various exposure environments; the loading rate is 0.05 MPa/s. In order to obtain the strain development along the CFRP sheet direction, five strain gauges are bonded on the CFRP surface and numbered as A, B, C, D, and E from left to right, as shown in Figure 6. A displacement meter is set at the bottom of the specimen midspan to obtain the deflection of the flexural specimen.

## 3. Results

### 3.1. Epoxy Resin Adhesive Sheet and CFRP Sheet

#### 3.1.1. Microstructure

**Figure 12:**
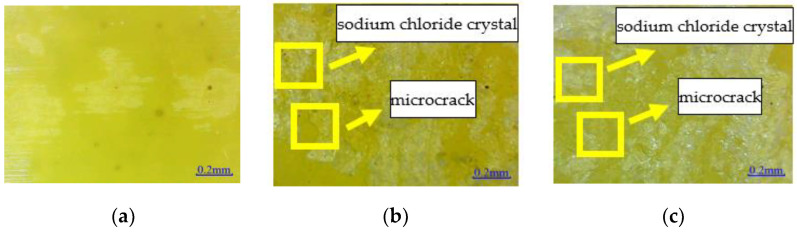
Microstructure: (**a**) EPND; (**b**) EPSI-400; and (**c**) EPSF-100.

The microstructure of epoxy resin is shown in Figure 12. The surface of EPND is smooth and clean, without any damage. A small amount of sodium chloride crystal is attached to the surface of EPSI-400, and there are small depressions and micro-cracks on the surface. However, many sodium chloride crystals are attached to the surface of EPSF-100, and noticeable depressions and microcracks are observed. The damage of epoxy resin in a salt-freeze coupling environment is more severe than that in a chlorine-salt immersion environment.

**Figure 13:**
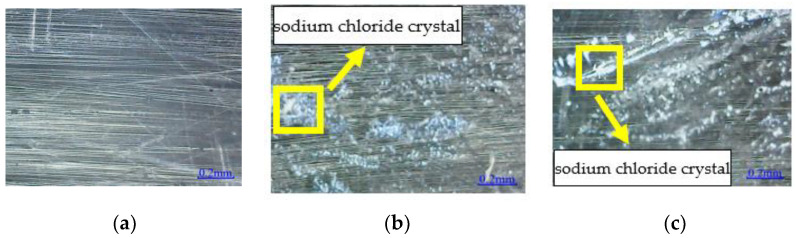
Microstructure: (**a**) CFRPND; (**b**) CFRPSI-400; and (**c**) CFRPSF-100.

Similarly, Figure 13 shows that the CFRPND surface is smooth and clean without damage. A small amount of sodium chloride crystal can be observed on the CFRPSI-400 surface and slight depressions and micro-cracks on the epoxy resin matrix. At the same time, the inner CFRPSI-400 fiber has no apparent damage. Many sodium chloride crystals are attached to the CFRPSF-100 surface, and the epoxy resin on the surface has noticeable depressions and microcracks, while the carbon fiber has no apparent damage. Compared with the chlorine immersion environment, the damage to the CFRP sheet in the salt-freeze coupling environment is more serious.

All these observations can be explained; the epoxy resin contained an ether bond due to a chemical reaction between resin and curing agent. Some uncured epoxy resins contain hydrophilic hydroxyl groups, and curing agents contain tertiary amines and a small amount of incomplete primary and secondary amines. These groups will be hydrolyzed to varying degrees in a salt solution, which leads to some depressions on the surface of epoxy resin and CFRP sheet. Furthermore, low temperature and chlorine ion environment can destroy the inner structure of epoxy resin, and the internal micro-cracks occur. The epoxy resin deterioration is derived from group hydrolysis and chloride ions erosion in the chlorine salt immersion environment. However, the coupled effect of the group hydrolysis, chloride ion erosion, and freeze–thaw cycles caused the damage to epoxy resin in the salt-freeze environment. In addition, the microcracks caused by freeze–thaw cycles promote the penetration of sodium chloride solution, leading to further damage to epoxy resin and CFRP sheet.

#### 3.1.2. Failure Mode

**Figure 14:**
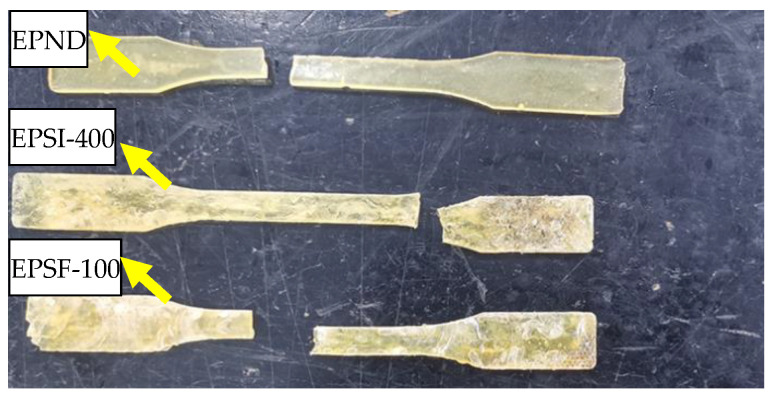
Failure mode of epoxy resin.

**Figure 15:**
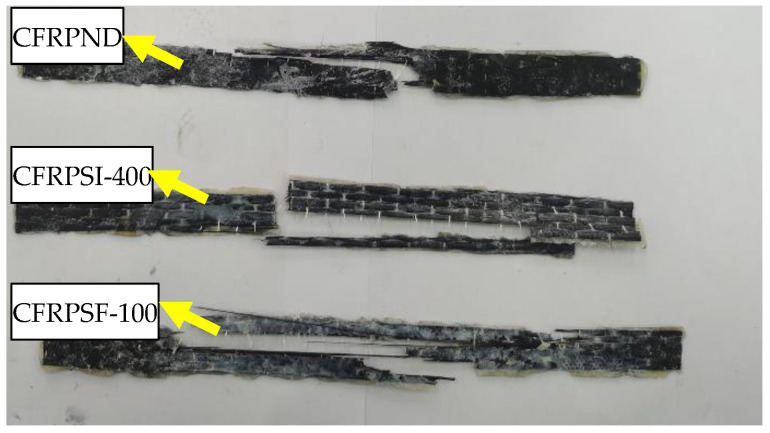
Failure mode of CFRP sheet.

The appearance and morphology of the epoxy resin and CFRP sheet are observed in the tensile test. Figure 14 shows the failure modes of EPND, EPSI-400, and EPSF-100, and there is no significant change at the initial loading stage. The specimens’ sudden breaks occur when the ultimate load is reached. The fracture of the epoxy resin appears randomly along with the standard interval, and the exposure environment has no significant influence on the failure pattern. The failure modes of CFRPND, CFRPSI-400, and CFRPSF-100 have brittle failures, as shown in Figure 15. There is no significant change in the initial loading stage. The epoxy resin on the surface of the CFRP sheet cracks with the progress of loading. The sample will break suddenly when the ultimate load is reached. The failure process of CFRP sheet is consistent with relevant research [12]. The fracture of CFRP sheet appeared randomly along with the standard interval, and the exposure environment has no significant influence on the failure pattern.

#### 3.1.3. Tensile Performance

**Figure 16:**
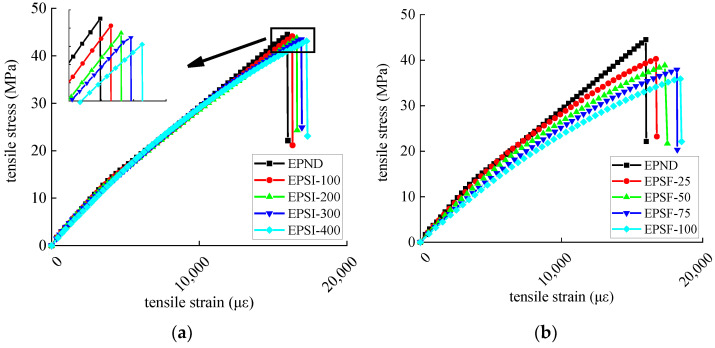
Stress–strain curve of epoxy resin: (**a**) chlorine salt immersion environment and (**b**) salt-freeze coupled environment.

**Figure 17:**
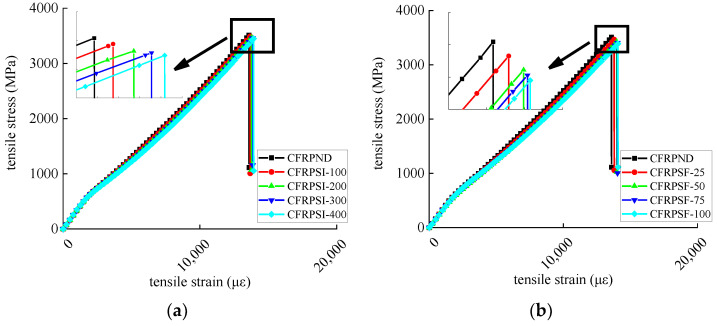
Stress–strain curve of CFRP sheet: (**a**) chlorine salt immersion environment and (**b**) salt-freeze coupled environment.

The tensile strength and ultimate tensile strain of epoxy resin and CFRP sheet are shown in Table 5 and Table 6. The change rate refers to the damage rate of the mechanical index of a deteriorated specimen relative to the non-deteriorated specimen. The tensile modulus of elasticity is shown in Table 7. The stress–strain relationship is shown in Figure 16 and Figure 17, respectively. The tensile strength of epoxy resin has a decreased trend, and the ultimate tensile strain has an increasing trend. The elastic modulus decreases, and the second half of the stress–strain curve is nonlinear, indicating that the mechanical properties of epoxy resin are seriously degraded in the exposed environment. The CFRP sheet exerts a similar deterioration trend for mechanical properties due to external epoxy resin protection. Compared with the chlorine immersion environment, the mechanical properties of epoxy resin and CFRP sheet degrade more seriously in the salt-freeze coupling environment. The tensile strength degradation rate of the CFRP sheet is lower than that of epoxy resin, indicating that the durability of the CFRP sheet is more robust than that of epoxy resin. With the increase in exposure time, the epoxy resin microcracks development from surface to inside is caused by the coupled effect of the group hydrolysis reaction, the freeze–thaw cycles, and chloride corrosion. In addition, epoxy resin is plasticized by water absorption in chloride solution, resulting in a nonlinear stress–strain curve. The mechanical properties of CFRP sheets are mainly determined by CFRP fiber because the tensile strength of epoxy resin is much lower than that of CFRP fiber. The damage to the epoxy resin matrix has little influence on the mechanical properties of CFRP sheets. The interface damage between carbon fiber and epoxy resin is the main factor leading to mechanical degradation of CFRP sheet, and the carbon fiber itself is not damaged.

### 3.2. Concrete Flexural Specimen

#### 3.2.1. Microstructure

**Figure 18:**
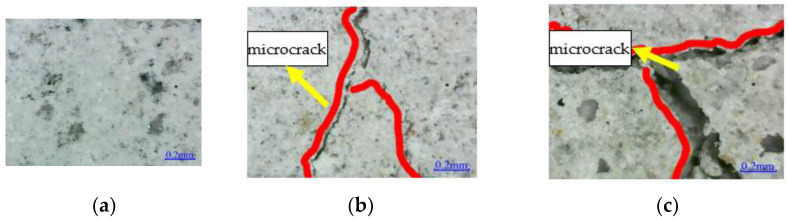
Microstructure of concrete surface: (**a**) URFND; (**b**) URFSI-400; and (**c**) URFSF-100.

**Figure 19:**
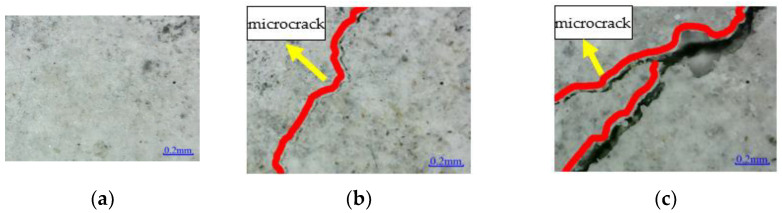
Microstructure of concrete surface: (**a**) RFND; (**b**) RFSI-400; and (**c**) RFSF-100.

The entire cement mortar matrix surface is observed for URFND and RFND specimens without environmental effect, as shown in Figure 18 and Figure 19. The surfaces of URFSI-400 and RFSI-400 have slight microcracks, while severe microcracks occur in URFSF-100 and RFSF-100. This can be explained by the crystallization extrusion pressure generated by the precipitation of sodium chloride crystals leading to microcracks in the specimen, and the microcracks development is positively related to the sodium chloride crystals. In addition, pore water continuously repeats the process of crystallization–liquefaction–crystallization under the effect of the freeze–thaw cycle. As a result, the inner crystals continuously produce frost heaving force and finally lead to the micro-cracks constantly produced under the repeated action of frost heaving force [6]. It indicates that the damage to specimens in a salt-freeze coupling environment is more severe than that in a chloride salt immersion environment.

**Figure 20:**
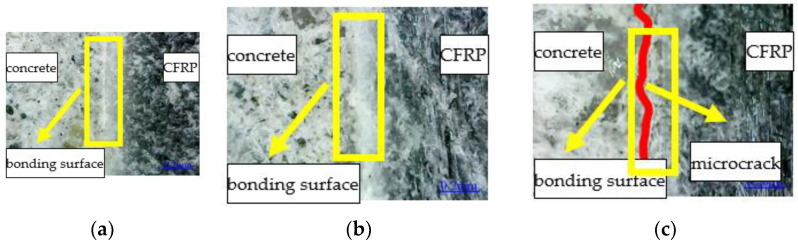
Microstructure of bonding surface between CFRP and concrete: (**a**) RFND; (**b**) RFSI-400; and (**c**) RFSF-100.

Figure 20 shows the microstructure of the bond surface between CFRP and concrete. No apparent damage is observed on the RFND and RFSI-400 interface, and micro-cracks appear on the bond surface of RFSF-100, indicating that the bond surface is more seriously damaged in the salt-freeze coupling environment than in the chlorine-salt immersion environment.

#### 3.2.2. Failure Mode

**Figure 21:**
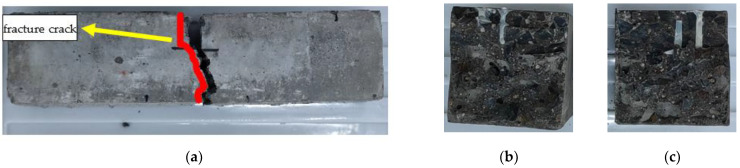
Failure mode of URFND: (**a**) side surface; (**b**) fracture surface 1; and (**c**) fracture surface 2.

**Figure 22:**
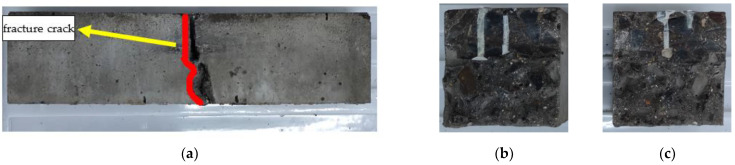
Failure mode of URFSI-400: (**a**) side surface; (**b**) fracture surface 1; and (**c**) fracture surface 2.

**Figure 23:**
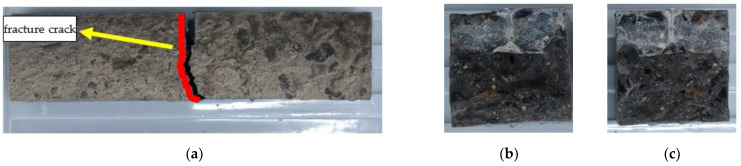
Failure mode of URFSF-100: (**a**) side surface; (**b**) fracture surface 1; and (**c**) fracture surface 2.

The failure mode of URFND is shown in Figure 21. Similarly, the failure mode of URFSI-400 is shown in Figure 22, and the failure mode of URFSF-100 is shown in Figure 23. The unreinforced specimen’s failure pattern had no noticeable change at the beginning of the loading process. Then, tiny cracks appear. The cracks develop rapidly, scaling up extends to the top of the specimen, and the failure pattern is normal section flexural damage.

**Figure 24:**
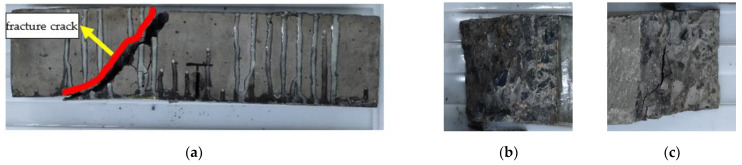
Failure mode of RFND: (**a**) side surface; (**b**) fracture surface 1; and (**c**) fracture surface 2.

**Figure 25:**
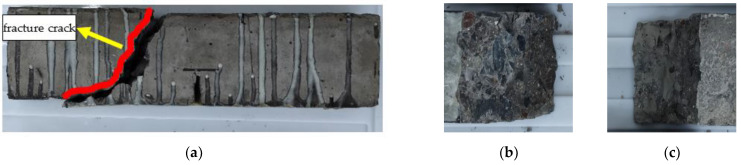
Failure mode of RFSI-400: (**a**) side surface; (**b**) fracture surface 1; and (**c**) fracture surface 2.

**Figure 26:**
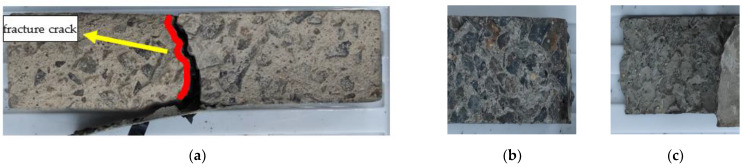
Failure mode of RFSF-100: (**a**) side surface; (**b**) concrete bonding surface; and (**c**) CFRP bonding surface.

The failure modes of the reinforced specimen are different in various exposure to environmental conditions. The failure mode is inclined section failure for RFND, and the upper and lower ends of the inclined crack are located near the loading and support points, respectively. As a result, the CFRP sheet and interface are not damaged, as shown in Figure 24. For specimen RFSI-400, its failure mode is similar to that of specimen RFND, as shown in Figure 25. Figure 26 shows the normal section bending failure for RFSF-100, and the interface between CFRP and concrete is completely debonding, leading CFRP flakes off the concrete surface.

For unreinforced bending specimens, the cracks initially occurred at the notch location (Figure 6) and then extension. The specimen’s upper side is subjected to compression, and the downside is subjected to tension. The stress is redistributed for CFRP reinforced concrete specimens because CFRP and concrete together into a whole load of engineering structures. The positive tensile stress at the inclined section is the largest, leading to its tensile failure. The bond strength between CFRP and concrete is reduced, and the strengthening effect is weakened, leading to the CFRP peeling off from the concrete surface after the salt-freeze coupling effect.

#### 3.2.3. Bending Performance

The bending strength of specimens can be calculated by Formula (1) as follows:

(1) $$f_{f}=\frac{Fl}{bh^{2}}$$

where *f_f_* is flexure strength (MPa); *F* is specimen failure load (N); *l* is distance between supports (mm), *l* = 300 mm; *b* is specimen width (mm), *b* = 100 mm; and *h* is specimen height (mm), *h* = 70 mm.

**Figure 27:**
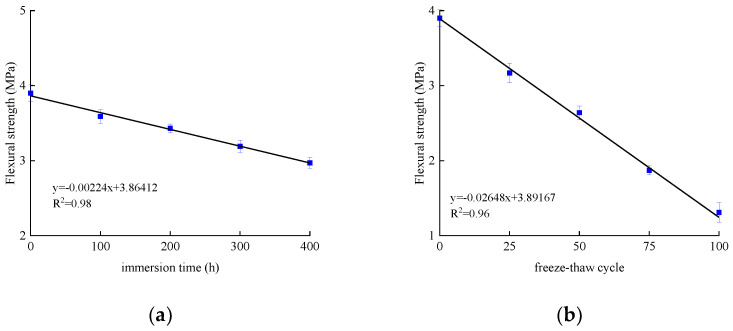
Flexural strength of unreinforced concrete specimen: (**a**) chlorine salt immersion environment and (**b**) salt-freeze coupled environment.

**Figure 28:**
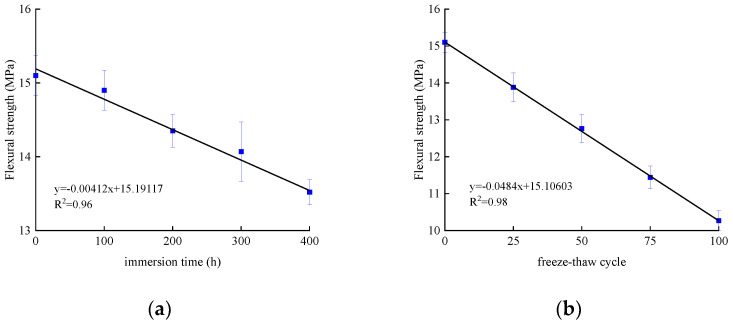
Flexural strength of reinforced concrete specimen: (**a**) chlorine salt immersion environment; and (**b**) salt-freeze coupled environment.

**Figure 29:**
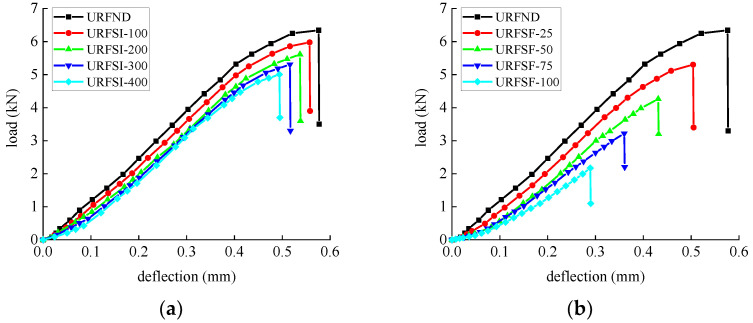
Load-deflection curve of unreinforced concrete flexural specimen: (**a**) chlorine salt immersion environment and (**b**) salt-freeze coupled environment.

**Figure 30:**
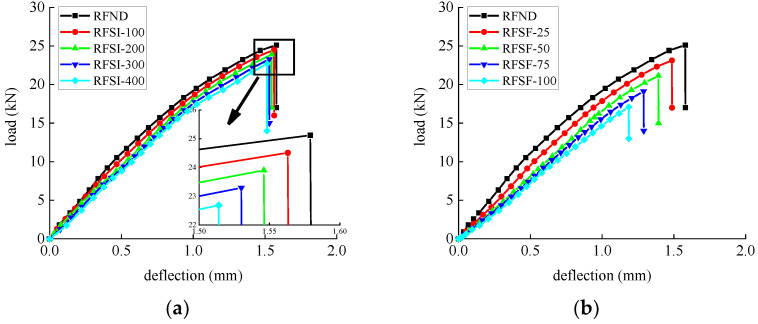
Load-deflection curve of reinforced concrete flexural specimen: (**a**) chlorine salt immersion environment and (**b**) salt-freeze coupled environment.

The flexural strength and deflection of specimens subjected to bending are shown in Table 8 and Table 9. The average value and standard deviation of the flexural strength of the test piece are shown in Figure 27 and Figure 28. Their load–deflection relationship is shown in Figure 29 and Figure 30, respectively. For unreinforced specimens, the standard deviation range of tensile strength is 0.06~0.13, the discrete type is small, and the data are relatively stable. For reinforced specimens, the tensile strength standard deviation range is 0.17~0.4. The discrete type is large, and the data stability is poor. The flexural strength of the strengthened specimens is 360% higher than that of the unreinforced specimens, which indicates that CFRP can effectively improve flexural strength. The bending strength, deflection, and compressive stiffness of all specimens decreased slightly after chlorine salt immersion, and the longer the immersion period, the more noticeable these changes occurred. The specimen micro-cracks are generated from surface to interior after being immersed in chlorine salt, and cracking continues to expand during the loading process until failure in advance, reducing the flexural strength stiffness and deflection of the specimen.

After the salt-freeze coupling effect, the unreinforced and strengthened bending specimens exert a similar decrease trend. The whole concrete structure becomes loose under the repeated action of frost heaving force, resulting in a higher reduction than in the chlorine salt environment. The bond force between CFRP and concrete is also the main factor affecting the mechanical properties of the flexural specimens. The degradation of the bond force reduces the bearing capacity of the flexural specimens in the salt-freeze coupling environment.

**Figure 31:**
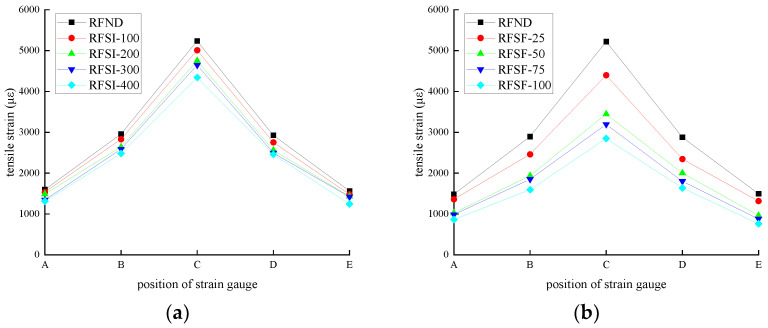
Strain of CFRP strip: (**a**) chlorine salt immersion environment and (**b**) salt-freeze coupled environment.

For CFRP reinforced flexural specimens, the tensile strain of CFRP strips at various positions is shown in Figure 31. The bond strength gradually decreases between CFRP and concrete due to the deterioration of epoxy resin with increasing chloride exposure time. Therefore, the tensile strength of CFRP decreases while the tensile strength of concrete increases, resulting in a decrease in the tensile strain of CFRP at various positions. Compared with a chlorine salt immersion environment, a salt-freeze coupling environment has more severe damage to CFRP and concrete bond performance.

### 3.3. Finite Element Analysis

#### 3.3.1. Epoxy Resin and CFRP Sheet

ABAQUS r2021x is used to simulate the tensile process of epoxy resin adhesive and CFRP sheet. The CFRP is regarded as a linear elastic material, and only the tensile stress in the fiber direction is considered in the finite element analysis. The CFRP is considered a failure when the CFRP reaches the ultimate tensile strain. There is no difference in damage form concerning different chlorine salt environments. Thus, the internal stress distribution and plasticizing effect can be ignored. The linear elastic model is selected to consider the limit tensile strength and the change in the chlorine salt environment. Similarly, the mechanical properties fitting curve equation is described above for CFRP and epoxy resin in a chloride environment, and the specific parameters of constitutive models are determined. The expressions are as follows:

(2) $$\sigma =\left\{\begin{array}{c} E_{CFRP}\varepsilon \;\varepsilon <\varepsilon_{CFRP}\\ 0\;\;\;\varepsilon >\varepsilon_{CFRP} \end{array}\right.$$

where *E_CFRP_* is modulus of elasticity for CFRP tension (MPa); and *ε_CFRP_* is ultimate tensile strain for CFRP.

(3) $$\sigma =\left\{\begin{array}{c} E_{EP}\varepsilon \;\varepsilon <\varepsilon_{EP}\\ 0\;\;\;\varepsilon >\varepsilon_{EP} \end{array}\right.$$

where *E_EP_* is modulus of elasticity for epoxy resin tension (MPa); and *ε_EP_* is ultimate tensile strain for epoxy resin.

**Figure 32:**
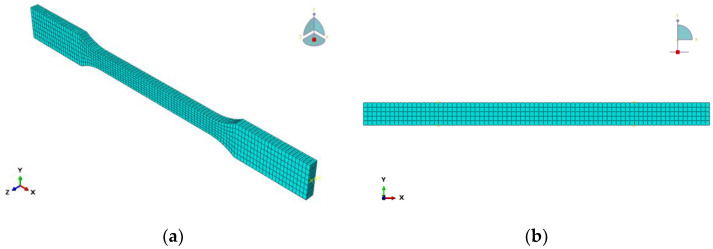
Mesh Dividing: (**a**) epoxy resin adhesive sheet and (**b**) CFRP sheet.

The epoxy resin finite element model is a three-dimensional solid structure, and the mesh division is shown in Figure 32a. The epoxy simulation element type is C3D8R (8-node hexahedron linear reduction integral solid element) and consists of 3570 units and 4944 nodes. Compared with the complete integral element, the C3D8R element contains only one integral point in the element’s center, and the solution result is more accurate and easier to converge.

The CFRP finite element model is a three-dimensional plane structure, and the mesh division is shown in Figure 32b. The element type is selected for S4R (4-node quadrilateral linear reduced-integral shell element) and consists of 385 elements and 468 nodes. The S4R element types can be used for modeling thin or thick shell structures, and it adopts a reduced-integral mode to guarantee stable performance.

Reinforcement sections are reserved at both ends of epoxy resin and CFRP models. In order to approach the actual situation, the reinforcement Section 1 is completely fixed in all six degrees of freedom (U1 = U2 = U3 = UR1 = UR2 = UR3 = 0). A reference point RP1 is set and coupled with the strengthened Section 2 to monitor the force value during the loading process, and a linear displacement of loading method is used for reference point RP1.

**Figure 33:**
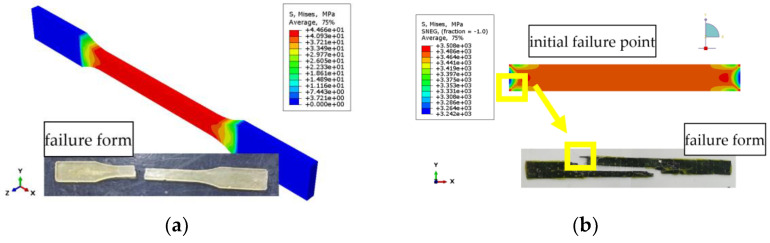
Stress nephogram: (**a**) Epoxy resin; (**b**) CFRP sheet.

The failure modes of epoxy resin in different environments are the same no matter the environmental effect. Figure 33a shows the stress distribution of EPND, and the maximum stress and strain are concentrated in the middle segment, which is consistent with the experimental result. Similarly, Figure 33b shows the stress distribution of CFRPND, and the maximum stress and strain first appeared in the gage section of four corner points. The stress and strain at the corner point first reach the ultimate tensile strength, resulting in split failure of the CFRP sheet, which is consistent with the experimental phenomenon.

**Figure 34:**
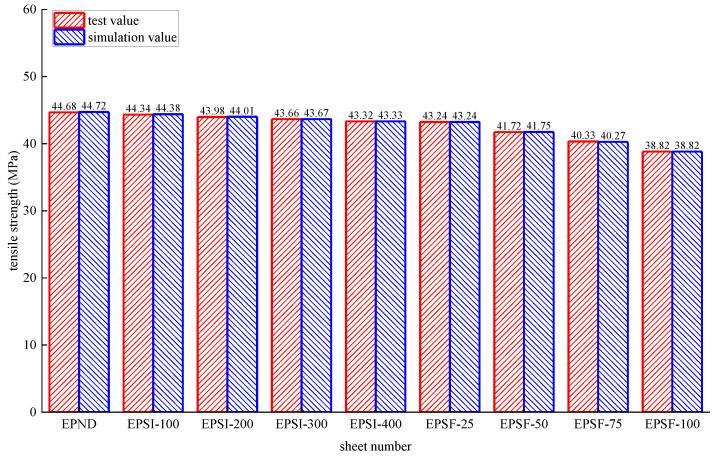
Test values and simulated values of tensile strength of epoxy resin.

**Figure 35:**
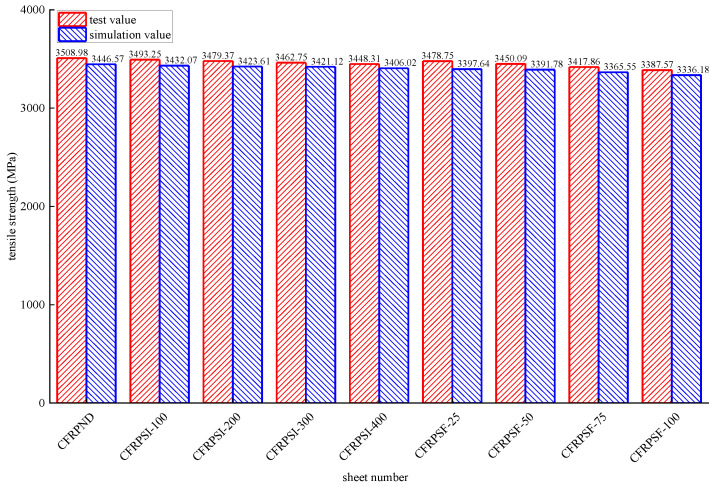
Test values and simulated values of tensile strength of CFRP sheet.

Figure 34 shows the experimental and simulated values of epoxy resin’s ultimate tensile strength subjected to different environmental effects. The maximum error between the simulated and experimental values is 1.24%, and the fitting effect is satisfactory. The maximum error between the simulated and experimental values is 1.77% for the CFRP sheet, as shown in Figure 35. The simulated value is slightly less than the test value because the fixture in the experimental test cannot ultimately hinder the transverse deformation at the corner of the sheet. However, a small transverse slip is occurred between the fixture and the CFRP strip, delaying the corner from reaching the ultimate strength.

#### 3.3.2. Concrete Flexural Specimen

According to ‘Concrete Structure Design’ (GB50010-2010), the CDP model and the determination method of concrete material parameters under uniaxial loading are used to simulate the mechanical behavior in this study. The stress–strain relationship of concrete under uniaxial tension and compression can be calculated by Equations (4) and (5), respectively. Furthermore, the concrete degradation law of mechanical properties is proposed under a salt-freeze coupling environment, and the concrete material is also simulated based on this law [24,25].

(4) $$\sigma_{t}=\left(1-d_{t}\right)E\varepsilon_{t}$$

(5) $$\sigma_{c}=\left(1-d_{c}\right)E\varepsilon_{c}$$

where *σ_t_* is concrete tensile stress; *ε_t_* is concrete tensile strain; *d_t_* is evolution parameters of concrete uniaxial tensile damage; *σ_c_* is concrete compressive stress; *ε_c_* is concrete ultimate compressive strain; *d_c_* is damage evolution parameters of concrete under uniaxial compression; and *E* is elastic modulus of concrete.

**Figure 36:**
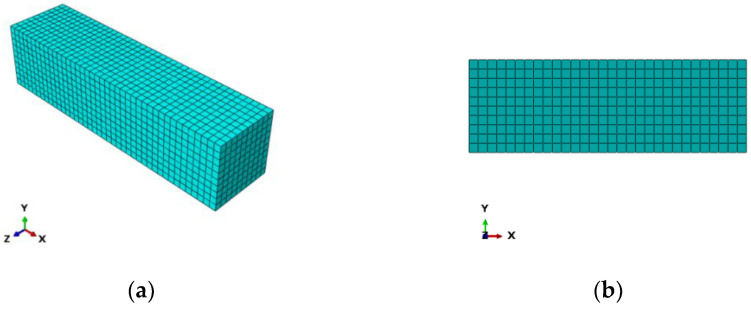
Mesh Dividing: (**a**) Concrete; (**b**) CFRP.

For the flexural specimen model of concrete, the constitutive relation of CFRP and the interface between CFRP and concrete are consistent with the axial compression specimen model. Therefore, the C3D8R solid element is used to simulate concrete material, and the S4R shell element is used to simulate CFRP material. The mesh division of concrete and CFRP is shown in Figure 36.

The ABAQUS software provides two methods for establishing a cohesion model based on traction–separation law: cohesive element and cohesive surface interaction method. Most studies often use the latter [26,27]. For traction–separation law, most studies adopt a two-line constitutive model, which is divided into a linear elastic segment before reaching ultimate strength and a linear decreasing stiffness segment after reaching ultimate strength. The stiffness and fracture energy expressions are listed as Equations (6) and (7). Damage initiation is related to traction–separation law, which reaches the moment of ultimate strength. An initial damage criterion suitable for lamination simulation of composites is provided by ABAQUS software and can be expressed with Equation (8):

(6) $$K=\frac{\tau }{\varepsilon }$$

(7) $$G=\frac{1}{2}\cdot \tau_{max}\cdot \varepsilon_{max}$$

where *K* is bond stiffness (N/mm^3^); *τ* is bond stress (MPa); *ε* is relative slip distance (mm); and *G* is fracture energy (N/mm).

(8) $${\left\{\frac{\varepsilon_{n}}{\varepsilon_{n}^{0}}\right\}}^{2}+{\left\{\frac{\varepsilon_{s}}{\varepsilon_{s}^{0}}\right\}}^{2}+{\left\{\frac{\varepsilon_{t}}{\varepsilon_{t}^{0}}\right\}}^{2}=1$$

where *ε_n_* is normal strain of bond layer; *ε_n_^0^* is limit normal strain of bond layer; *ε_s_* and *ε_t_* are tangential strain of bond layer; and *ε_s_^0^* and *ε_t_^0^* are limit tangential strain of bond layer.

The degradation law of the bond force between CFRP and concrete in different environments is shown in Equations (9) and (10).

(9) $$\tau_{It}=0.993^{t}\cdot \tau$$

(10) $$\tau_{FTn}=0.987^{n}\cdot \tau$$

where *τ* is bond strength without deterioration (MPa); *τ_It_* is bond strength after *t* hours of chlorine salt immersion (MPa); and *τ_FTn_* is bond strength after *n* times of salt freeze coupling (MPa).

The underlying block’s four degrees of freedom (Y, Z, RXY, and RYZ) are fixed to keep the loading scheme consistent with the experiment. Similarly, the upper block is coupled with the reference point RP-1, and all five degrees of freedom (X, Y, RXY, RYZ, and RZX) of the reference point are fixed to obtain output load and displacement. Hard contact and no friction are used between concrete and the loading point. The reference points of the above two loading points are applied concentrated linear force in the Z direction, which is converted into a stress rate of 0.05 MPa/s.

**Figure 37:**
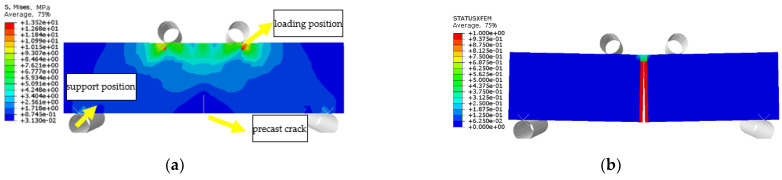
URFND: (**a**) stress nephogram and (**b**) damage nephogram of prefabricated crack.

**Figure 38:**
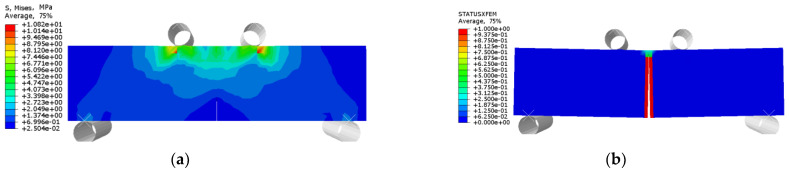
URFSI-400: (**a**) stress nephogram and (**b**) damage nephogram of prefabricated crack.

**Figure 39:**
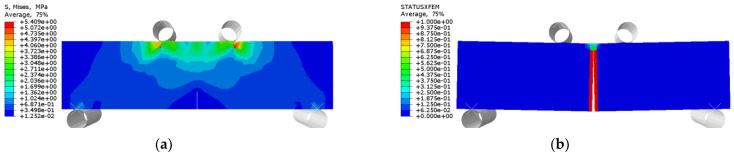
URFSF-100: (**a**) stress nephogram and (**b**) damage nephogram of prefabricated crack.

**Figure 40:**
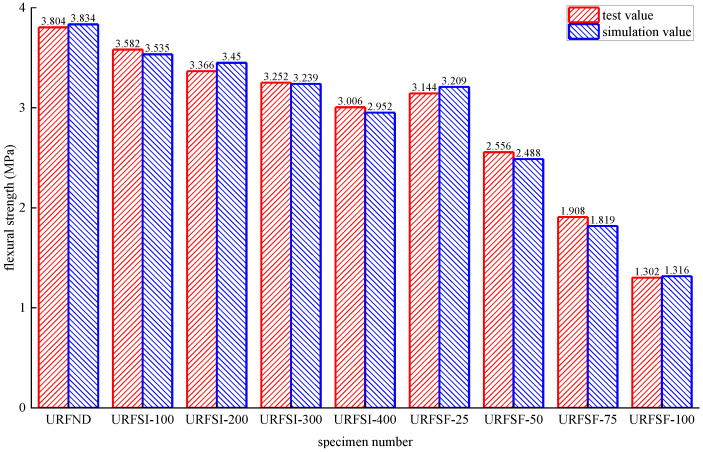
Test values and simulated values of flexural strength of unreinforced concrete flexural specimen.

The stress and crack damage distribution of URFND, URFSI-400, and URFSF-100 are shown in Figure 37, Figure 38 and Figure 39, respectively. The maximum stress is concentrated in the normal section area at the prefabricated crack. The stress distribution trend of specimens subjected to bending has no significant change in the chloride salt environment, and the only difference is the stress value. The failure modes of specimens are prefabricated cracks that develop upward to the top of the beam, and the normal section is subjected to bending. The experimental and simulation data of unreinforced concrete specimens under bending in different environments are shown in Figure 40. The maximum error between simulated and experimental values is 4.66%, indicating an ideal fitting effect.

**Figure 41:**
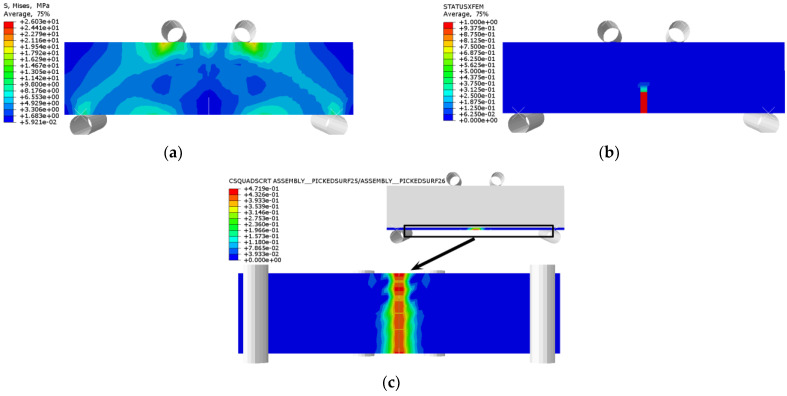
RFND: (**a**) stress nephogram; (**b**) damage nephogram of prefabricated crack; and (**c**) damage nephogram of bonding surface.

**Figure 42:**
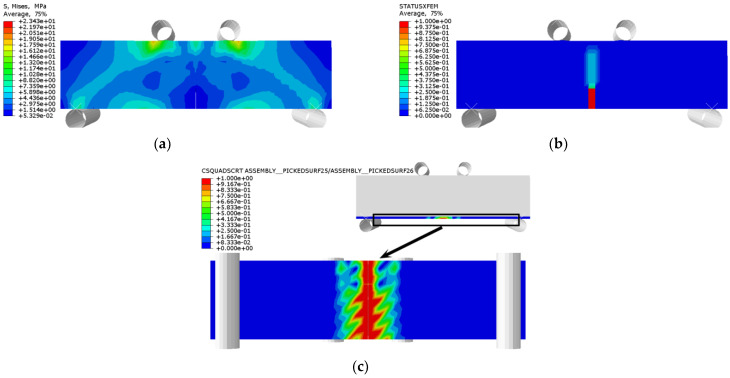
RFSI-400: (**a**) stress nephogram; (**b**) damage nephogram of prefabricated crack; and (**c**) damage nephogram of bonding surface.

**Figure 43:**
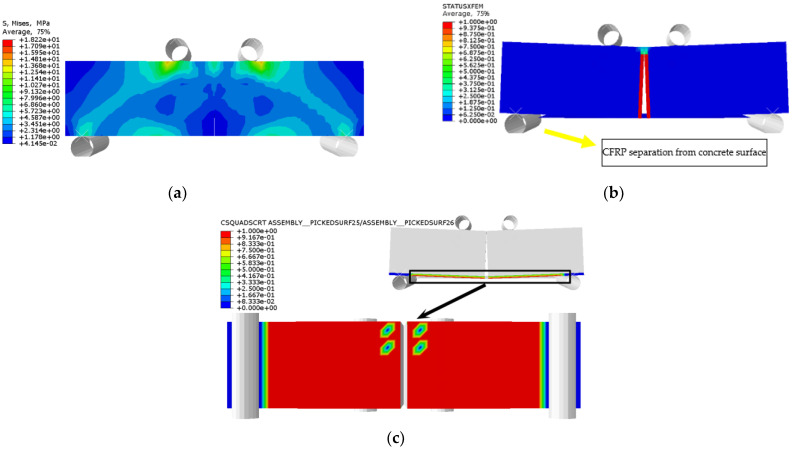
RFSF-100: (**a**) stress nephogram; (**b**) damage nephogram of prefabricated crack; and (**c**) damage nephogram of bonding surface.

**Figure 44:**
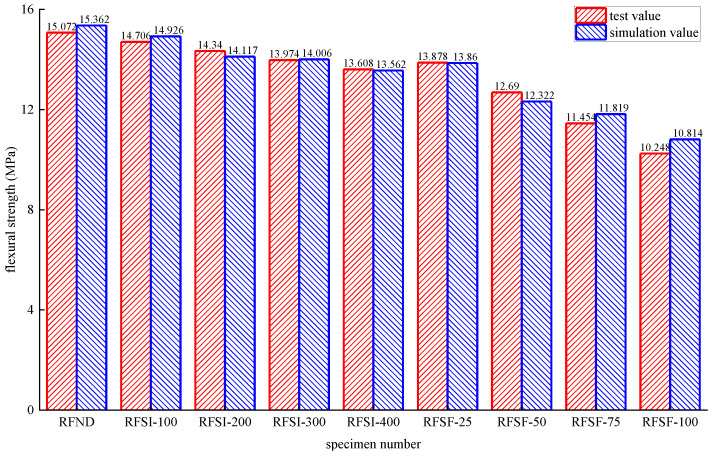
Test values and simulated values of flexural strength of reinforced concrete flexural specimen.

The stress distribution, prefabricated crack damage, and CFRP-concrete interface bond damage of RFND, RFSI-400, and RFSF-100 are shown in Figure 41, Figure 42 and Figure 43, respectively. For the specimens RFND and RFSI-400, the maximum stress is located in the line area between the upper and lower pressure points, while the prefabricated crack does not extend upward. The bonding surface only had weak damage at the prefabricated crack, which could be ignored, indicating that the failure is located in the concentration area of the maximum stress. For the specimen RFSF-100, although the maximum stress is located in the connecting area of the upper and lower pressure points, the prefabricated crack develops upward to the top of the beam, which is the flexural failure of the normal section. The damage to the bonding surface on the whole tensile side reaches 1, which represents the bonding force completely fails, and CFRP falls off from the concrete surface. The experimental test and simulation values of the reinforced bending specimens in different environments are shown in Figure 44. The maximum error between simulation and test values is 5.52%.

## 4. Conclusions

(1) Groups of the hydrolysis reaction and the erosion of chloride ions causes the apparent structure damage of epoxy resin and CFRP sheet. The coupling effect of chloride ion erosion and the freezing-thawing cycle leads to the further aggravation of the damage to the sheet. The apparent structural damage directly leads to the decrease of the tensile property of the sheet. The tensile strength exerts a higher degradation rate in the salt-freeze coupling environment when compared with the chlorine salt immersion environment. For epoxy resin, the degradation rate of tensile strength in a salt-freeze coupling environment is 327% higher than that in a chloride salt environment, while the value is 78% for the CFRP sheet.
(2) The finite element model of the tensile mechanical model is developed for epoxy resin and CFRP. The simulated and experimental values for tensile strength have a good agreement; the maximum error of epoxy resin is only 1.24%, and of CFRP is only 1.77%.
(3) Concrete is damaged by the crystallization extrusion pressure caused by the precipitation of sodium chloride crystal in a chlorine immersion environment. The specimens’ damage in the salt-freeze coupling environment is severe, and the flexural strength degradation rate is higher than in the chlorine salt immersion environment. For the specimens without reinforcement, the flexural strength degradation rate in the salt-freeze coupling environment is 187% higher than that in the chlorine-salt immersion environment. However, the degradation rate reaches 202% for the reinforcement specimens.
(4) The degradation law of bond performance between CFRP and concrete is discussed, and a finite element mechanical model is established. The maximum error between simulation and experimental data is only 5.52%, and the failure mode is basically consistent with the test data. It can be explained that the CFRP improved the tension capacity of the specimen, which inhibits the development of prefabricated cracks and improves the bearing capacity.

## Figures and Tables

**Table 1:** Tensile properties of CFRP and epoxy adhesive.

Material	Tensile Strength/MPa	Elastic Modulus/GPa	Elongation at Break/%
CFRP composite	3520	267	1.78
Epoxy resin adhesive	54.3	2.7	2.25

**Table 2:** Concrete mix proportion.

Material	Water	Cement	Sand	Crushed Stone
content/(kg/m^3^)	209	387	635	1169

**Table 3:** Number of epoxy resin and CFRP sheet.

Exposure Environment	Epoxy Resin Adhesive Sheet	CFRP Sheet
No deterioration	EPND	CFRPND
Chlorine salt immersion for 100 h	EPSI-100	CFRPSI-100
Chlorine salt immersion for 200 h	EPSI-200	CFRPSI-200
Chlorine salt immersion for 300 h	EPSI-300	CFRPSI-300
Chlorine salt immersion for 400 h	EPSI-400	CFRPSI-400
Salt-freezing coupling 25 times	EPSF-25	CFRPSF-25
Salt-freezing coupling 50 times	EPSF-50	CFRPSF-50
Salt-freezing coupling 75 times	EPSF-75	CFRPSF-75
Salt-freezing coupling 100 times	EPSF-100	CFRPSF-100

**Table 4:** Number of concrete specimens.

Exposure Environment	Unreinforced Specimen	Reinforced Specimen
No deterioration	URFND	RFND
Chlorine salt immersion for 100 h	URFSI-100	RFSI-100
Chlorine salt immersion for 200 h	URFSI-200	RFSI-200
Chlorine salt immersion for 300 h	URFSI-300	RFSI-300
Chlorine salt immersion for 400 h	URFSI-400	RFSI-400
Salt-freezing coupling 25 times	URFSF-25	RFSF-25
Salt-freezing coupling 50 times	URFSF-50	RFSF-50
Salt-freezing coupling 75 times	URFSF-75	RFSF-75
Salt-freezing coupling 100 times	URFSF-100	RFSF-100

**Table 5:** Tensile strength of epoxy resin and CFRP sheet.

Epoxy Resin Adhesive Sheet	Tensile Strength/MPa	Change Rate/%	CFRP Sheet	Tensile Strength/MPa	Change Rate/%
EPND	44.68	—	CFRPND	3508.98	—
EPSI-100	44.34	−0.76	CFRPSI-100	3493.25	−0.45
EPSI-200	43.98	−1.57	CFRPSI-200	3479.37	−0.84
EPSI-300	43.66	−2.28	CFRPSI-300	3462.75	−1.32
EPSI-400	43.32	−3.04	CFRPSI-400	3448.31	−1.73
EPSF-25	43.24	−3.22	CFRPSF-25	3478.75	−0.86
EPSF-50	41.72	−6.62	CFRPSF-50	3450.09	−1.68
EPSF-75	40.33	−9.74	CFRPSF-75	3417.86	−2.6
EPSF-100	38.82	−13.12	CFRPSF-100	3387.57	−3.46

**Table 6:** Tensile strain of epoxy resin and CFRP sheet.

Epoxy Resin Adhesive Sheet	Tensile Strain/με	Change Rate/%	CFRP Sheet	Tensile Strain/με	Change Rate/%
EPND	15,992	—	CFRPND	13,616	—
EPSI-100	16,402	2.56	CFRPSI-100	13,703	0.64
EPSI-200	16,499	3.17	CFRPSI-200	13,778	1.19
EPSI-300	16,811	5.12	CFRPSI-300	13,868	1.85
EPSI-400	17,046	6.59	CFRPSI-400	13,954	2.48
EPSF-25	16,602	3.81	CFRPSF-25	13,740	0.91
EPSF-50	17,614	10.14	CFRPSF-50	13,847	1.7
EPSF-75	18,205	13.84	CFRPSF-75	13,972	2.61
EPSF-100	18,883	18.08	CFRPSF-100	14,095	3.52

**Table 7:** Tensile modulus of elasticity of epoxy resin and CFRP sheet.

Epoxy Resin Adhesive Sheet	Tensile Modulus of Elasticity/MPa	Change Rate/%	CFRP Sheet	Tensile Modulus of Elasticity/MPa	Change Rate/%
EPND	2554	—	CFRPND	256,622	—
EPSI-100	2482	−2.82	CFRPSI-100	256,122	−0.19
EPSI-200	2474	−3.13	CFRPSI-200	255,632	−0.39
EPSI-300	2421	−5.21	CFRPSI-300	253,048	−1.39
EPSI-400	2325	−8.97	CFRPSI-400	250,471	−2.4
EPSF-25	2455	−3.88	CFRPSF-25	254,066	−1
EPSF-50	2282	−10.65	CFRPSF-50	252,053	−1.78
EPSF-75	2048	−19.81	CFRPSF-75	248,353	−3.22
EPSF-100	1903	−25.49	CFRPSF-100	244,681	−4.65

**Table 8:** Flexural strength of concrete flexural specimen.

Unreinforced Specimen	Flexural Strength/MPa	Change Rate/%	Reinforced Specimen	Flexural Strength/MPa	Change Rate/%
URFND	3.804	—	RFND	15.072	—
URFSI-100	3.582	−5.836	RFSI-100	14.706	−2.428
URFSI-200	3.366	−11.514	RFSI-200	14.34	−4.857
URFSI-300	3.252	−14.511	RFSI-300	13.974	−7.285
URFSI-400	3.006	−20.978	RFSI-400	13.608	−9.713
URFSF-25	3.144	−17.35	RFSF-25	13.878	−7.922
URFSF-50	2.556	−32.808	RFSF-50	12.69	−15.804
URFSF-75	1.908	−49.842	RFSF-75	11.454	−24.005
URFSF-100	1.302	−65.773	RFSF-100	10.248	−32.006

Note: $\mathrm{Change}\;\mathrm{rate}=\frac{F_{ND}-F_{D}}{F_{ND}}\times 100\%$, *F_ND_*: flexural strength of non-deteriorated specimen; *F_D_*: flexuralstrength of deteriorated specimen.

**Table 9:** Deflection of concrete flexural specimen.

Unreinforced Specimen	Deflection/mm	Change Rate/%	Reinforced Specimen	Deflection/mm	Change Rate/%
URFND	0.576	—	RFND	1.579	—
URFSI-100	0.557	−3.299	RFSI-100	1.563	−1.013
URFSI-200	0.537	−6.771	RFSI-200	1.546	−2.09
URFSI-300	0.514	−10.764	RFSI-300	1.53	−3.103
URFSI-400	0.494	−14.236	RFSI-400	1.514	−4.117
URFSF-25	0.505	−12.326	RFSF-25	1.491	−5.573
URFSF-50	0.431	−25.174	RFSF-50	1.392	−11.843
URFSF-75	0.359	−37.674	RFSF-75	1.285	−18.619
URFSF-100	0.289	−49.826	RFSF-100	1.185	−24.953

Note: $\mathrm{Change}\;\mathrm{rate}=\frac{D_{ND}-D_{D}}{D_{ND}}\times 100\%$, *D_ND_*: deflection of non-deteriorated specimen; *D_D_*: deflection of deteriorated specimen.

## Data Availability

All data generated or analyzed during this study are included in this published article.
